# Mental Nerve Neurectomy: A Comparison of Titanium Screw Versus Gutta-Percha Oburation of the Mental Foramen

**DOI:** 10.7759/cureus.56426

**Published:** 2024-03-18

**Authors:** Syed Ammar Yasir, Ali Akhtar Khan, Sana Somair, Ramsha Nawaz, Hamza Asif, Saadat Ullah

**Affiliations:** 1 Department of Oral and Maxillofacial Surgery, Armed Forces Institute of Dentistry, Rawalpindi, PAK; 2 Department of Oral and Maxillofacial Surgery, Combined Military Hospital, Rawalpindi, PAK; 3 Department of Orthodontics, Armed Forces Institute of Dentistry, Rawalpindi, PAK

**Keywords:** pain remission, titanium screw, gutta percha sticks, neurectomy, trigeminal neuralgia

## Abstract

Objective: This study aims to compare the two mental foramen obturating techniques in parameters like operator's ease, postoperative displacement of obturation material, and remission of pain after peripheral neurectomy in patients with trigeminal neuralgia.

Study design: This study adopts a single-centered comparative analytical approach.

Place and duration of study: This study was performed in the Department of Oral and Maxillofacial Surgery, Armed Forces Institute of Dentistry (AFID), from October 1, 2023, to February 10, 2024.

Methodology: Sixty patients fulfilling the inclusion criteria were included and divided into two groups (30 each), and peripheral neurectomy was performed under local anesthesia. In one group, gutta-percha sticks were used to obturate the foramina, and in the other group, titanium screws were used. Postoperatively, the operator’s ease, postoperative displacement, and relapse of pain were recorded.

Results: Titanium screws were easy to handle, and there was no relapse of pain because of no displacement postoperatively when compared with gutta-percha sticks, which were hard to manipulate and showed a relapse of pain.

Conclusions: Titanium screws have better postoperative outcomes when compared with gutta-percha sticks in terms of the operator's ease, relapse of pain, and postoperative displacement.

## Introduction

Of the 1.7 million known sufferers of trigeminal neuralgia, 26% have been reported to commit suicide as a result of severe pain, which is why it is referred to as the *suicide Disease*.

By definition, neuralgia is a pain in a nerve pathway, which itself is not an ailment but a symptom of some other illness [1]. It can be due to any infection, trauma, or a degenerative nerve disease [2]. The orofacial region has three types of neuralgia: trigeminal neuralgia (12 out of every 100,000 people) [3], occipital neuralgia (3 out of every 100,000 people) [4], and post-herpetic neuralgia (10% of patients having shingles) [5]. Trigeminal neuralgia can be classified into ophthalmic (6%), maxillary (39%), or mandibular (55%) types based on the involved nerve division [6]. Moreover, mandibular trigeminal neuralgia is subdivided into inferior alveolar nerve neuralgia, lingual nerve neuralgia, and mental nerve neuralgia.

Trigeminal neuralgia can occur in individuals of any age, although it is more commonly observed in females. Statistics indicate a ratio of 3:7 in favor of females. And among females, older ones are affected more commonly. Postmenopausal women from 40 to 70 years of age have a greater prevalence of trigeminal neuralgia [7]. Symptoms of trigeminal neuralgia involve sudden stabbing and shocking pain in the dermatome supplied by the branch involved [8]. The pain is very severe and can be easily aggravated by a stimulus as low as an air blow. There are two types of trigeminal neuralgia: Type 1, characterized by sharp and sudden pain, and Type 2, where the pain is more constant.

Historically, multiple treatment modalities have been introduced to treat trigeminal neuralgia. Generally, these can be divided into conservative, moderately invasive, and surgical management. Conservative modalities include medical management by medications like carbamazepine [9], oxcarbazepine [10], tricyclic antidepressants, phenytoin, and anticonvulsants [11]. In some patients, multi-drug regimens can be utilized for effective management of this ailment. Moderately invasive procedures involve rhizotomy [12], a procedure to remove or destroy the nerve fibers causing problems. There are different types of rhizotomy. Mainly they include chemical rhizotomy (glycerol rhizotomy), radiofrequency ablation, and endoscopic rhizotomy. Surgical management involves neurectomy [13] or neurovascular decompression [14] from the peripheral branches up to the pons from where the trigeminal nerve originates (Jannetta Procedure) [15].

Specifically, for neuralgia of the mental nerve, mental nerve neurectomy is a successful treatment modality because of the clear visibility of nerve bundles emerging from the mental foramen. This procedure is performed under local anesthesia, and it involves a neurectomy of the nerve fibers and blocking the mental foramen with a biocompatible material to avoid the regeneration of diseased nerve fibers. Commonly, bone wax, gutta-percha cones [16], bone chips, and stainless steel screws are used to obturate the mental foramen. These materials are difficult to manipulate during surgery and have a tendency to displacement postoperatively, leading to poor blockade of regenerative nerve fibers as in the case of gutta-percha cones or bone chips. Some of them are a bit less biocompatible like in the case of stainless steel.

Our study aimed to compare the effectiveness of gutta-percha cones and titanium screws in blocking the mental foramen after neurectomy, considering variables such as postoperative recurrence (remission of pain), postoperative dislodgement of obturation material, and perioperative operator's ease of use. This study will provide a baseline for further research to evaluate the effectiveness of titanium screws and gutta-percha cones to block the mental foramen after a mental nerve neurectomy. Moreover, it can help in setting a foundation for titanium screw usage in the obturation of other foramina in the management of nerve dysesthesias. 

## Materials and methods

This prospective analytical comparative study was conducted in the Department of Oral and Maxillofacial Surgery, Armed Forces Institute of Dentistry (AFID), from January 10, 2023, to October 10, 2023.

Patient recruitment and study groups

After receiving ethical committee permission from the ethical review board of AFID (registry number 918/Trg, May 13, 2020), the study enrolled 60 subjects (30 in each group). The World Health Organization (WHO) Sample Size calculator was used to calculate sample size (level of significance was kept 5 and power of test 80). Patients were declared having trigeminal neuralgia based on the classic history of paroxysmal pain, which was intense, sharp, shooting, or stabbing in nature, lasting for seconds to minutes, and easily provoked by stimuli like touching of the dermatome or clenching of teeth. Surprisingly, only patients with type 1 neuralgia reported to the department in the said period. Diagnostic block, that is, local anesthesia in the region of the mental foramen was used to confirm the diagnosis.

Patients of both genders within age limits of 40-70 years having unilateral pain with typical signs of neuralgia were included in the study. Moreover, they had a history of medical management of trigeminal neuralgia with carbamazepine (600-1,200 mg). There was no odontogenic or any other organic cause of pain. Patients having atypical facial pain or pain of unknown origin or having a bilateral (uncommon) distribution of pain were excluded from the study. Patients having obvious odontogenic or organic causes of pain were also excluded.

Patients fulfilling inclusion criteria who chose neurectomy (surgical severing or removal of nerve fibers) as a treatment option for said disease were divided into two groups (a consecutive nonprobability sampling technique was used for randomization). Postneurectomy, the mental foramina were blocked using gutta-percha sticks in one group and titanium screws in the other group. The success of the procedure was defined based on the operator’s ease, recurrence of neuralgia (remission of pain), and displacement of obturating media after surgery. After obturating the foramina, the wound was cleaned thoroughly with normal saline, and closure was done with 3-0 Vicryl. All the procedures were performed by the same operator in the same setup. Patients were followed up at regular intervals, specifically every three months, and were assessed for the recurrence of neuralgia (i.e., pain) and displacement of the obturating medium (evaluated on orthopantomogram [OPG]).

Surgical method

**Figure 1:**
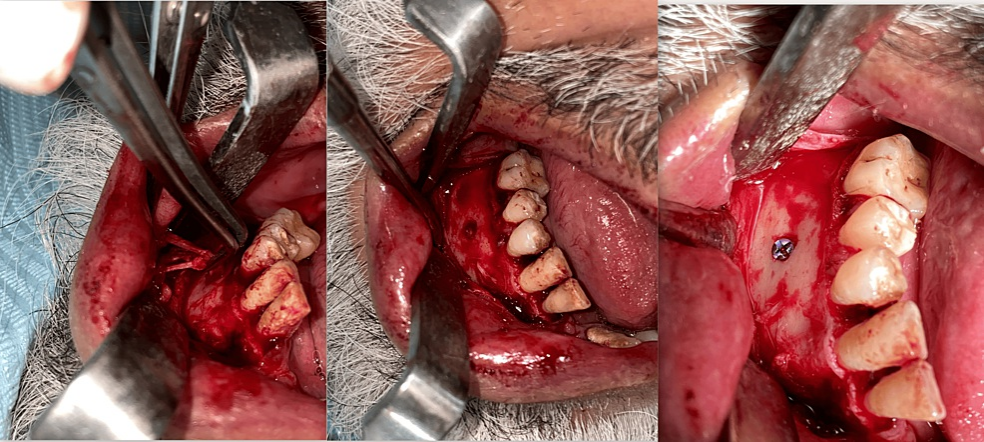
Neurectomy followed by titanium screw obturation (Group A).

The surgical procedures were performed under local anesthesia and with appropriate medication. The treatment plan was explained to the patients, and their consent was obtained before the procedure. Under the best possible aseptic conditions, local anesthesia was administered. The mental nerve was exposed after raising the mucoperiosteal flap using a crevicular incision from the first molar to the central incisor on the affected side. The mental foramen was located, and skeletonization of nerve fibers was performed. A neurectomy was done after releasing the branches of the mental nerve and pulling the nerve out of the foramen. A titanium screw was used for the obturation of the mental foramen in Group A (Figure 1). 

**Figure 2:**
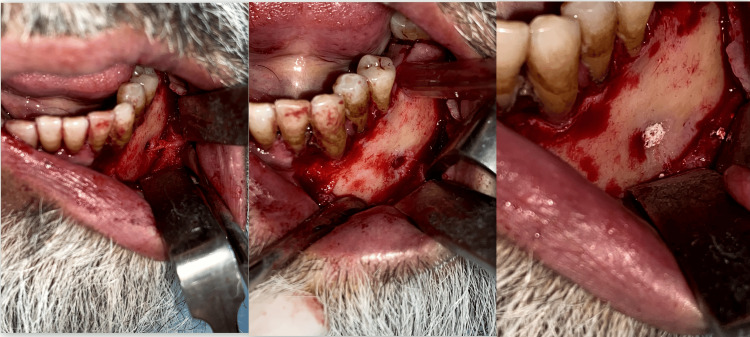
Neurectomy followed by gutta-percha obturation (Group B).

In Group B, mental foramen obturation was done using gutta-percha sticks (Figure 2).

Data collection

Assessment of the *operator’s ease* was done immediately after the procedure subjectively on the Likert scale. The operator was asked to mark a level from 1 to 5, where level 1 showed a very easy procedure and level 5 showed a very difficult procedure.

The *recurrence of pain* was assessed using a visual analog scale ranging from 0 to 10, where 0 represented no pain and 10 represented the worst possible pain. The patient was asked to mark a point according to the perceived pain. It was assessed after three months of the procedure. Table 1 shows different ranges used for pain assessment.

**Table 1:** Different ranges of a visual analog scale.

Range score	Intensity of pain
0	No pain
1-3	Mild pain
4-7	Moderate pain
8-10	Severe pain

**Figure 3:**
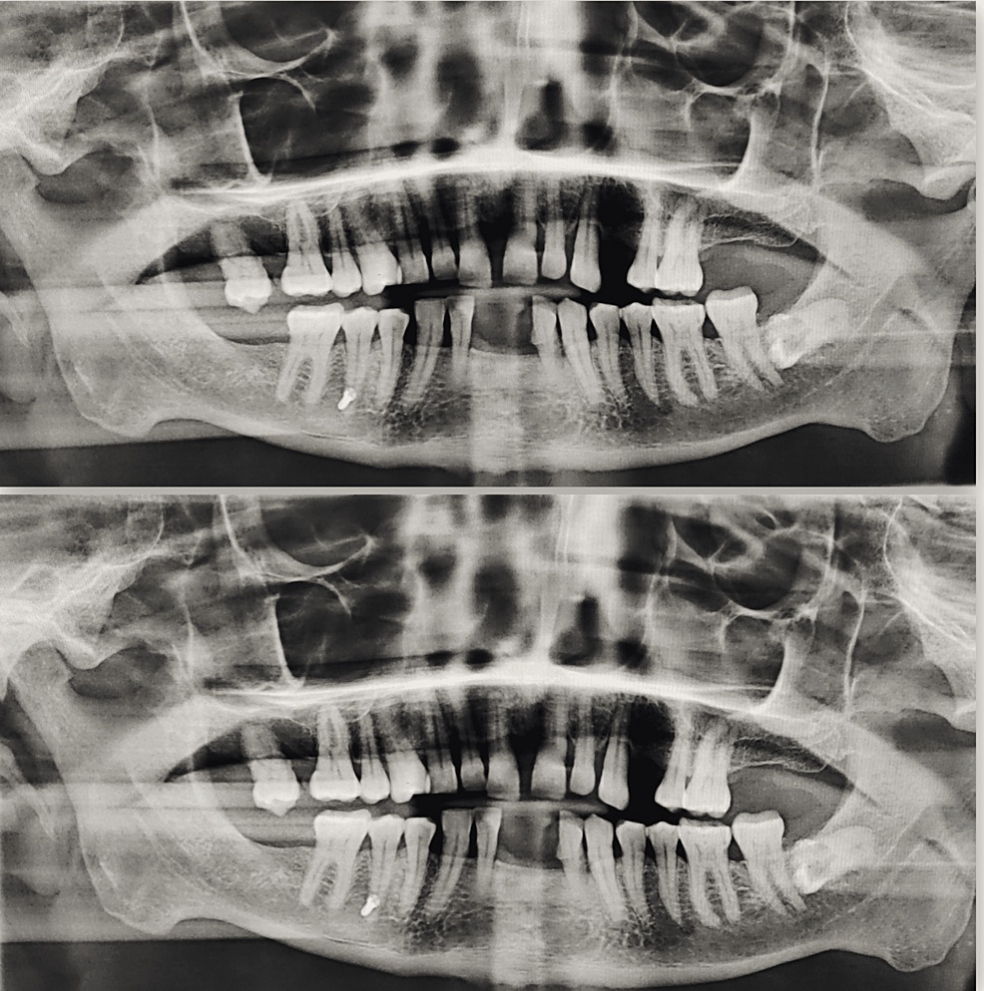
Titanium screw position after one month (above) and three months (below).

The *displacement of obturating material* was assessed by the operator visually on the patient’s OPG after three months of the procedure. Figure 3 shows the undisplaced titanium screw at one month and three months intervals in Group A.

**Figure 4:**
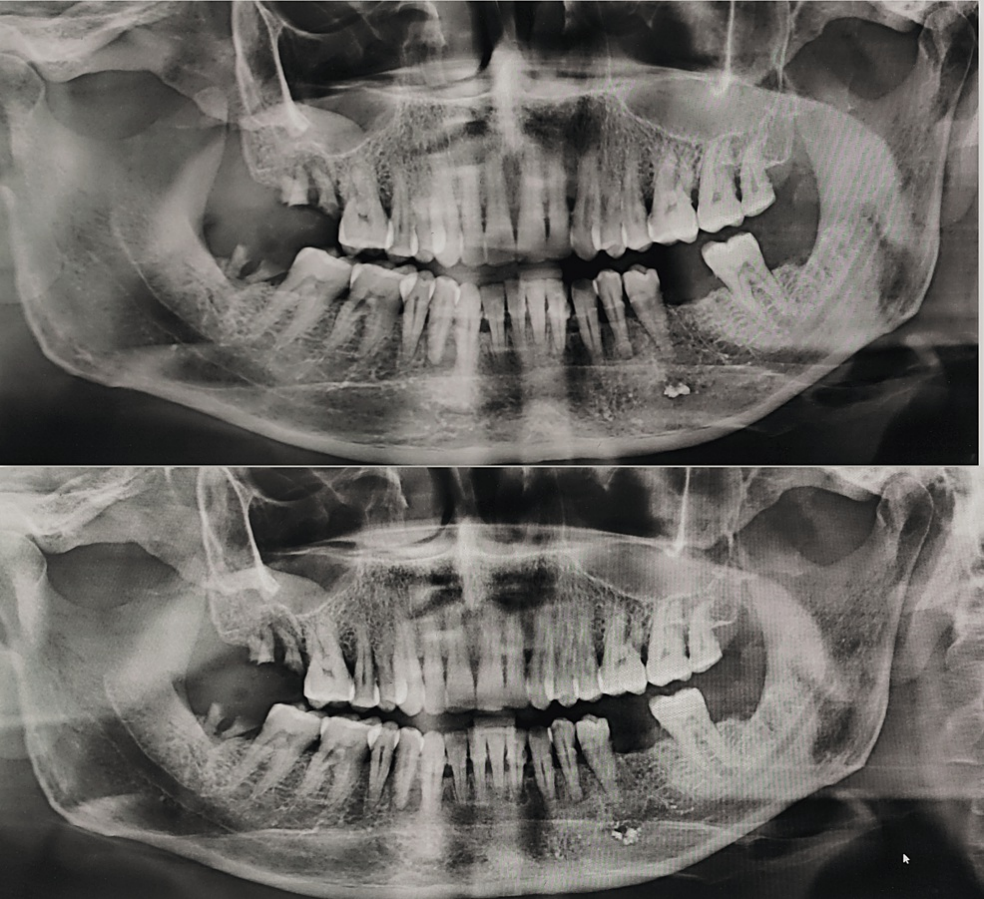
Gutta-percha position after one month (above) and three months (below).

Figure 4 shows the displacement of obturation material in Group B.

Statistical analysis

Statistical analysis was done by using IBM SPSS Statistics for Windows, Version 23.0 (IBM Corp., Armonk, NY). Quantitative and Qualitative variables were analyzed. Quantitative variables were expressed as mean and standard deviation (SD), and qualitative variables were expressed as frequencies and percentages.

For qualitative variables such as gender, operator’s ease, relapse of pain, and displacement of obturating material, a chi-square test was used for two groups. For comparison of quantitative variables such as the age of patients, an independent sample t-test was used, and for level of significance, the *P*-value was used. A *P*-value of <0.05 was considered statistically significant.

## Results

The study enrolled 60 patients, comprising 20 males and 40 females. The mean age of the participants was 51 years (Table 2).

**Table 2:** Age and gender of patients.

Parameters	Titanium screw (*n *= 30)	Gutta-percha (*n *= 30)
Age (Years)	51.40 ± 5.09	51.20 ± 5.84
Gender		
Male	9 (30%)	11 (37%)
Female	21 (70%)	19 (63%)

Excellent operator ease was noted in the titanium screw obturation method (Group A), with 12 obturations rated as very easy, 15 as easy, and 3 as difficult. Compromised results were observed in the gutta-percha obturation method (Group B), with four obturations rated as very easy, 12 as easy, six as difficult, and eight as very difficult (Table 3).

**Table 3:** Comparison of the operator's ease, relapse of pain, and displacement of obturating material between two groups. *P*-value was calculated using the chi-square test.

Parameters	Titanium screw (*n *= 30)	Gutta-percha (*n *= 30)	*P*-value
Operator’s ease			
Very easy	12 (40%)	4 (13%)	0.004
Easy	15 (50%)	12 (40%)	
Difficult	3 (10%)	6 (20%)	
Very difficult	0 (0%)	8 (27%)	
Relapse of pain			
No pain	23 (77%)	8 (27%)	<0.001
Mild pain	7 (23%)	12 (40%)	
Pain	0 (0%)	8 (27%)	
Moderate pain	0 (0%)	2 (15%)	
Displacement of obturating material			
Displacement	0 (0%)	20 (66%)	<0.001
No displacement	30 (100%)	10 (34%)	

In Group A, 23 patients observed no remission of pain, while seven experienced mild pain after three months. In Group B, eight patients had no pain, 12 had mild pain, eight had marked pain, and two had severe pain of a neuralgic nature after three months (Table 3).

On the visual assessment of OPG, no obturating material was displaced in Group A, while 20 cases of displacement were observed in Group B after three months (Table 3).

## Discussion

Traditionally, trigeminal neuralgia was managed by medicaments like carbamazepine, gabapentine, tricyclic antidepressants, or new formulations like oxcarbazepine. In their meta-analysis, Zhao and Ge compared the efficacy of carbamazepine and gabapentine. They found carbamazepine to be superior in the treatment of trigeminal neuralgia [17]. However, they stated that like other medications, carbamazepine has adverse effects like ataxia and dizziness or drowsiness; therefore, proper monitoring is required. These medications are not definitive and lose their efficacy after a certain time and, hence, are accompanied by many complications [18]. More invasive methods include rhizotomy, cryotherapy [19], and thermal coagulation, with glycerol rhizotomy [20] being a frequently performed procedure. Young [21] stated that glycerol rhizotomy loses its efficacy over time and often necessitates multiple sessions. Moreover, it carries complications like local tissue toxicity, burning alcohol neuritis, and fibrosis in the region. Other treatment modalities include microvascular decompression, balloon decompression [22], gamma knife surgery, or the Jannetta procedure. However, these are considered highly invasive, and patients are often reluctant to choose any of these options.

Peripheral neurectomy is a modern management of trigeminal neuralgia, which is considered a much safer option because neurectomy is performed in direct vision [23]. However, owing to the regenerative potential of the trigeminal nerve, the nerve fibers regenerate, resulting in a relapse of pain after the surgery [24]. To avoid this, the foramen through which the fibers project out needs to be blocked. In mental nerve peripheral neurectomy, the mental foramen can be blocked by any material that fits the size of the foramen. The material should be biocompatible and easy to use and should not be displaced after being placed in the foramen.

Perioperative ease is one of the main aspects of any surgical procedure. Relapse of pain frequently occurs after a neurectomy, and displacement of obturating material after seizing the mental foramen is a known complication following mental nerve neurectomy. Despite using titanium screws frequently in the orofacial region, their utilization for mental foramen obturation is not a very common practice. In literature, few studies are available in which stainless steel (only one publication) or gutta-percha is used to obturate the mental foramen after mental nerve neurectomy. 

To the best of our knowledge, no such comparative study has been done or published in the literature to date. Patients with chronic trigeminal neuralgia were involved in our study in which conservative management modalities such as medication (carbamazepine) or glycerol rhizotomy lost their efficacy. Furthermore, all the procedures were performed by one surgeon in a comparable operation time. Patient baseline characteristics were almost similar in all patients so the difference among groups can be attributed to the efficacy of titanium screw and gutta-percha.

Our study suggests that using a titanium screw for obturation of the mental foramen after peripheral neurectomy of the mental nerve is a better approach than using gutta-percha for its obturation. The titanium screw is easy to use, prevents relapse of pain, and does not displace from the foramen if fixed appropriately. This study will hopefully provide a foundation for a more detailed analysis of titanium screw obturation of the mental foramen after peripheral neurectomy.

Limitations

Limited sample size and absence of placebo are two major limitations of this study.

## Conclusions

A titanium screw is an effective obturation material for the mental foramen, which can be successfully employed after mental nerve neurectomy when compared with gutta-percha.
